# Seismic Assessment of RC Bridge Columns Retrofitted with Near-Surface Mounted Shape Memory Alloy Technique

**DOI:** 10.3390/ma13071701

**Published:** 2020-04-05

**Authors:** Ammar Abbass, Reza Attarnejad, Mehdi Ghassemieh

**Affiliations:** School of Civil Engineering, College of Engineering, University of Tehran, Tehran P.O. BOX 4563-11155, Iran; ammarabbass@ut.ac.ir (A.A.); m.ghassemieh@ut.ac.ir (M.G.)

**Keywords:** shape memory alloys, near-surface mounted, retrofit RC bridge columns, self-centering

## Abstract

From past earthquakes, it has been found that the large residual displacement of bridges after seismic events could be one of the major causes of instability and serviceability disruption of the bridge. The shape memory alloy bars have the ability to reduce permanent deformations of concrete structures. This paper represents a new approach for retrofitting and seismic rehabilitation of previously designed bridge columns. In this concept, the RC bridge column was divided into three zones. The first zone in the critical region of the column where the plastic hinge is possible to occur was retrofitted with near-surface mounted shape memory alloy technique and wrapped with FRP sheets. The second zone, being above the plastic hinge, was confined with Fiber-Reinforced Polymer (FRP) jacket only, and the rest of the column left without any retrofitting. For this purpose, five types of shape memory alloy bars were used. One rectangular and one circular RC bridge column was selected and retrofitted with this proposed technique. The retrofitted columns were numerically investigated under nonlinear static and lateral cyclic loading using 2D fiber element modeling in *OpenSees* software. The results were normalized and compared with the as-built column. The results indicated that the relative self-centering capacity of RC bridge piers retrofitted with this new approach was highly greater than that of the as-built column. In addition, enhancements in strength and ductility were observed.

## 1. Introduction

It is observed that the majority of seismic design or retrofitting guidelines for RC bridges have focused to ensure the ductile behavior of the bridge under seismic loads and to prevent failure under exceptionally strong earthquakes [1,2]. This philosophy makes the bridge columns suffer from extensive damage and then large residual deformation will appear in the bridge columns and subsequently the service of the bridge will be interrupted. Thus, based on latest researches that have been conducted on the behavior of bridges after the earthquakes, the assessment and mitigation of residual deformations of the bridge columns remain one of the principal issues which needs to be deeply discussed [3].

On the other hand, different materials and techniques have been used to enhance the seismic behavior of old designed concrete bridge columns. Several researchers have experimentally investigated the effect of Fiber-Reinforced Polymer (FRP) jacketing on the RC bridge columns performance and the results showed that the ductility capacity and energy dissipation have improved [4,5,6]. However, despite the advantages of this method, notable residual deformations were observed, which highly affect the serviceability of the bridge after the earthquake [7,8,9].

Recently, near-surface mounted (NSM) strengthening technique was developed to enhance the flexural strength of RC columns [10,11,12]. In this method, steel or Fiber-Reinforced Polymer (FRP) rods or strips are inserted and glued by means of grout or epoxy resin into pre-cut grooves along the column. However, the debonding failure mode and lack of ductility under cyclic loading of NSM-FRP strengthened column have been observed [12,13,14]. Therefore, the NSM-FRP technique in combination with externally bonded FRP jacketing has been conducted to investigate the flexural and/or shear behavior of several bridges and building columns [12,15,16,17,18,19]. Most of the previous studies conducted on strengthening the bridge columns with the NSM-FRP technique have focused only on the flexural strengthening of the RC columns. To the authors’ knowledge, few studies discussed the permanent (residual) deformation parameter of strengthened columns with the NSM-FRP technique. Ding et al. [15] found that the NSM-Basalt FRP (BFRP) technique combined with BFRP jacketing has a significant effect on both strengthening and ductility of the strengthened columns. However, they noticed that the residual displacement increased by increasing the bonding length of the BFRP bars. Fahmy et al. [16] compared lap-spliced RC columns retrofitted using (NSM-BFRP) technique with well-designed columns (WDC). The results demonstrated that this retrofitting technique can remarkably improve the hysteretic response of RC columns with a lap-splice deficiency; but compared with an un-retrofitted lap-spliced RC column, the residual drifts of the retrofitted lap-spliced RC columns were not improved significantly.

Based on the aforementioned studies, the NSM-FRP technique faces the critical problem of residual deformations of the strengthened RC columns. Therefore, the main objective of this paper is that if RC bridge columns are retrofitted in such a way that they can sustain large displacement with minimum residual drifts after the ground motion, and then not only the strength capacity of the bridge will be increased but also the functionality of the bridge after the earthquake will be insured. 

To address large residual drifts shortcoming and achieve a good level of strength and ductility capacity in the RC bridge columns, NSM strengthening technique includes using innovative Shape Memory Alloys (SMAs) bars with FRP jacketing is proposed in this paper. This is because the unique characteristics of SMAs which have the ability to undergo large strains and then return to their initial state when it is heated which is called the shape memory effect or the stress is removed which is called the superelastic effect [20]. These novel characteristics motivated researchers to investigate their potential applications in civil engineering fields. As examples, some applications of SMAs in civil structures can be found in [21,22,23,24,25,26].

Recent studies have been focused on strengthening RC beam with NSM technique using SMA bars, especially iron-based Shape Memory Alloy (Fe-SMA). In the study by Shahverdi et al. [27], the flexural responses and service level of RC retrofitted beams were improved. However, to the knowledge of the authors, there is no research to-date regarding the use of NSM-SMA technique to retrofit RC bridge columns.

In this study, an innovative NSM-SMA with FRP jacket technique to retrofit RC bridge columns was proposed. Four types of superelastic SMAs in addition to one shape memory effect alloy used in civil engineering fields have been selected for use in this retrofitting technique. Moreover, this paper presents a comparative numerical study by using Open System for Earthquake Engineering Simulations (*OpenSees*) software of the performance of two columns retrofitted using the proposed retrofitting technique.

## 2. Research Significance 

Shape Memory Alloy (SMA) reinforcement bars in the plastic hinge regions have emerged as the design of new bridge columns and beam-column joints [3,28]. However, according to the authors’ knowledge, so far, all the previous experimental and numerical studies associated with SMA reinforcement for bridge columns have focused on replacing flexural steel bars with SMA bars in the plastic hinge region of the RC columns but there is almost no study about feasibility using SMA bars with the NSM technique to retrofit RC bridge columns [3,29,30]. 

The novel retrofit technology proposed in this paper is based on using SMA bars as NSM reinforcement and an external FRP jacket the critical regions of RC bridge columns in which the plastic hinge is possible to occur. Moreover, while other conventional retrofitting and strengthening techniques have interested in the flexure and shear behavior of retrofitted RC columns, the new proposed technique in this paper, in addition to enhancing the load carrying and ductility capacity, focused on mitigating the residual drifts of the retrofitted RC bridge columns.

## 3. Shape Memory Alloy

Nitinol or NiTi SMA alloys that possess several desirable properties such as large recoverable strains, hysteretic damping, and excellent corrosion resistance, have been widely used in civil engineering fields [31]. However, the high cost of NiTi SMAs made their use in large scale structures such as RC bridge restricted. Therefore, researchers have developed various Fe-based and Cu-based low cost SMAs. Fe-based SMAs show good workability, machinability, weldability, and wide transformation hysteresis as compared to NiTi SMAs [32]. Tanaka et al. [33] presented a ferrous polycrystalline SMA (Fe-Ni-Co-Al-Ta-B) which shows a very high deformability capacity of over 13% at room temperature. Omori et al. [34] developed a new Fe-based SMA (FeMnAlNi) which has the same superelasticity of NiTi alloy but smaller temperature dependence of the superelastic stress. Araki et al. [35] produced and tested another low cost (Cu-based) SMAs bars. They found that CuAlMn SMAs have higher machinability than NiTi SMAs, as well as a high potential used in seismic applications. More recently, the Swiss Federal Laboratories for Materials Science and Technology (Empa) in Switzerland has developed a novel Fe-based (FeMnSi) SMA [36,37,38]. Moreover, the potential applications of this SMA in strengthening RC beams have been experimentally studied [27,39].

Many different types of SMAs have been used as longitudinal reinforcements in the plastic hinge region of the RC bridge columns [3,29,40,41]. While other researchers used active SMA spiral confinement technique to retrofit RC bridge column, Andrawes et al. [42] experimentally and analytically studied RC bridge columns confined with active NiTi SMA spiral and Carbon Fiber-Reinforced Polymer (CFRP) sheets. The results showed that the average residual drift of columns retrofitted with this technique was about 41% smaller than that of the as-built columns. Shin and Andrawes [9] also experimentally compared active SMA spiral confinement technique in the plastic hinge zone of RC bridge columns with other traditional passive techniques. The results confirmed that the ductility capacity and energy dissipation capability of the columns retrofitted with active SMA spirals significantly increased.

**Figure 1:**
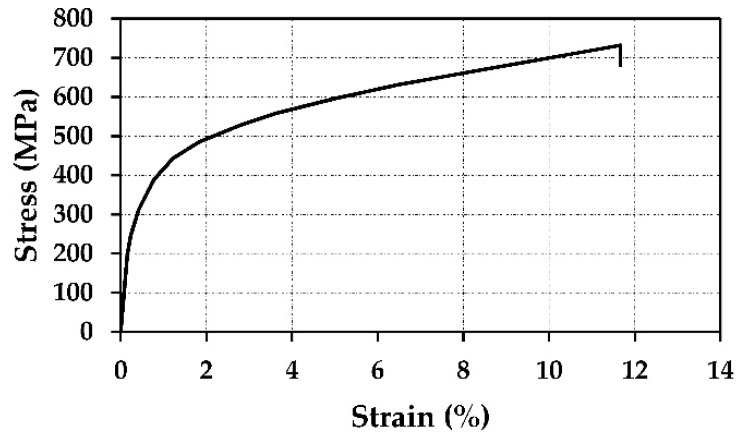
Stress-strain curve of FeMnSi SMA [27].

Four types of superelastic SMAs, in addition to one shape memory effect alloy used in civil engineering fields, have been selected in this study. The mechanical properties of four superelastic SMA bars are obtained from [30] and listed in Table 1. Figure 1 also shows the mechanical properties of one shape memory effect alloy bar adopted from. [27]. Although SMAs do not show yielding process, the term yield refers to the initiation of phase transformation of SMA and the yield strain was calculated by defining the austenite to Martensite starting stress $f_{y}$ by the elastic modulus (*E*) [30].

## 4. Numerical Investigation of Bridge Columns

### 4.1. Bridge Model Description

**Figure 2:**
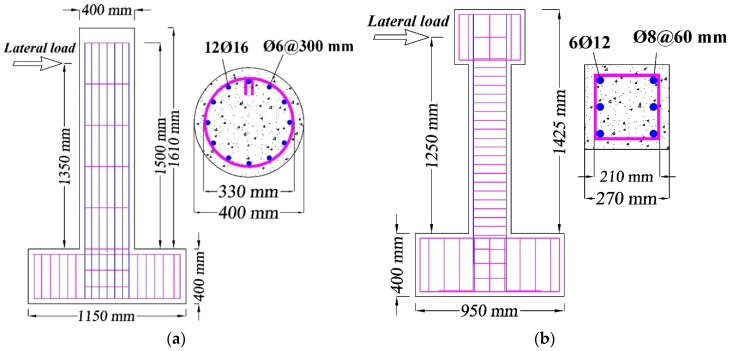
Schematic of geometry and reinforcement details of two selected columns: (**a**) Kawashima et al. column [45]; (**b**) Ding et al. column [15].

In this study, the numerical investigations were conducted on two reference tested scaled RC bridge columns, one was a circular cross-section and the other was rectangular. The analytical bridge model of the circular column was adopted from the experimental study of Kawashima et al. [45]. In their study, the hysteretic behavior of CFRP wrapped columns was discussed. While the RC rectangular cross-section column tested by Ding et al. [15] was used to build the analytical model of the rectangular column, they tested several RC columns retrofitted with NSM-BFRP and BFRP jacketing technique. The circular and rectangular cross-section bridge columns had an effective height of 1350 mm and 1250 mm, respectively. Two axial loads of 188 and 206 kN were lumped on the top of circular and rectangular columns. The schematic drawings of the two bridge columns with dimensions and reinforcement details are illustrated in Figure 2. As illustrated in Figure 2, the diameter of the circular cross-section column is 400 mm and the thickness of the concrete cover is 35 mm, while the dimensions for the rectangular cross-section column are 270 mm × 270 mm and for the concrete cover is 30 mm.

Table 2 also describes the material properties and dimensions of the tow bridge columns adopted in this study.

### 4.2. Proposed Retrofitting Technique

**Figure 3:**
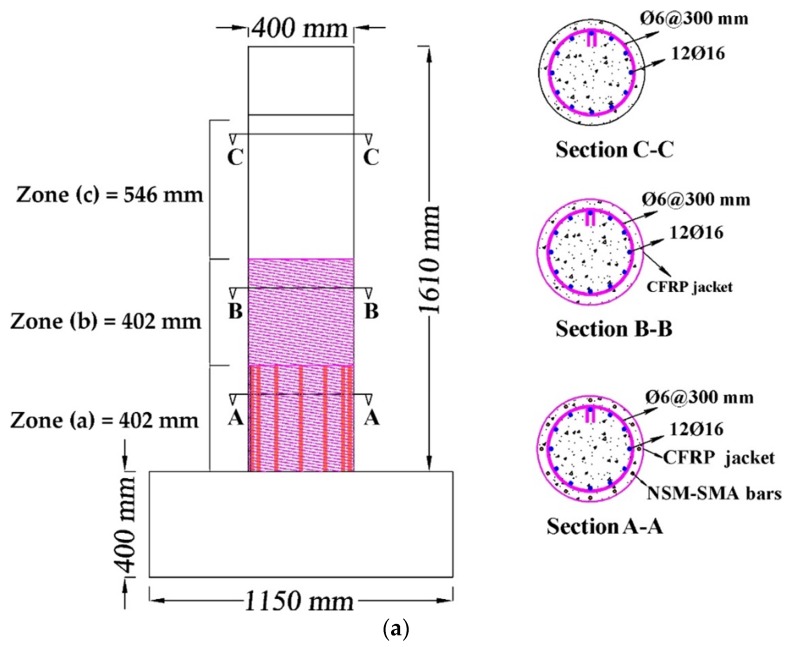

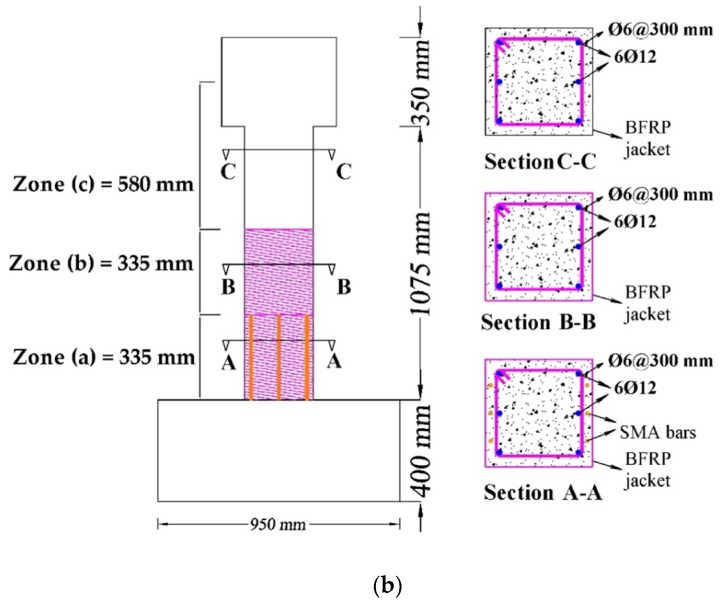
Schematic showing the proposed retrofitting technique: (**a**) Circular cross-section column; (**b**) rectangular cross-section column.

The efficiency of using the SMA bars as NSM reinforcements combined with FRP sheet jackets to retrofit and improve the seismic behavior of RC bridge columns was investigated analytically in this paper. The finite element program *OpenSees* (version 2.4.3) was used in the study. In the proposed retrofitting technique, as illustrated in Figure 3, each column is divided into three zones. The first zone (a), where the plastic hinge of the bridge column is possible to occur, was retrofitted with the NSN-SMA technique and with FRP sheet jackets. To prevent the damage in the zone (b), it was only wrapped with the same volumetric ratio of FRP jackets that were in the first zone. The rest of the bridge column zone (c) remained without any retrofit.

Five types of SMA bars and two types of FRP jackets were used in this study. For the rectangular cross-section bridge column, three layers with 0.111 mm thickness of BFRP jackets were applied, while one layer of CFRP with 0.111 mm thickness was used for circular cross-section bridge column. To show the effectiveness of using NSM-SMA bars and to make a logical comparative study, the thickness and number of layers of the two types of FRP jackets were selected similar to those in the original tests. The mechanical properties of the two types of FRP jackets are shown in the Table 3.

Based on above-mentioned, the bridge columns were designated as “a-b-c” where “a” represents the geometric of the bridge column such as “C” (circular cross-section) or “R” (rectangular cross-section), “b” shows the type of FRP sheet “B” (Basalt Fiber-reinforced polymers) or “C” (Carbon Fiber-reinforced polymers), and “c” references the type of NSM-SMA bars (SMA1, 2, 3, 4, and 5). R-0-0 and C-0-0 represent as-built rectangular cross-section and circular cross-section columns, respectively. All studied columns in this paper divided into two groups based on the two references tested columns. Table 4 shows as-built and retrofitted columns of the two groups.

According to previous researches conducted on strengthening and retrofitting RC columns with NSM bars technique, there are no clear limitations for the ratio of NSM bars. Some of these researches are specified in Table 5. However, based on Caltrans 2010 [46], the maximum allowable reinforcement ratio of RC bridge columns equals to 4%. Since the SMAs as shown in Table 1 have different mechanical properties, different diameters of SMAs bars were used. In this study, for all retrofitted columns in Group2, the number of NSM-SMA bars were 6, placed on each of two opposite sides of the column. While, 12 NSM-SMA bars distributed along the circumference of the RC columns were used to retrofit columns in the Group1. The diameter of NSM NiTi45 bars was 10mm, the diameter of NSM FeNCATB and FeMnSi-based bars was 12mm, the diameter of NSM CuAlNi bars was 14mm, and of FeMnAlNi was 12 mm. The number and diameter of NSM-SMAs bars were selected in such a way that the all retrofitted RC columns had almost the same axial forces.

The plastic hinge length, $L_{P}$, for the rectangular cross-section bridge columns was calculated according to the relation given by Yuan et al. [48]; as follows:

(1) $$L_{P}{=L}_{P0}{+L}_{Pf}=\left(0.08L+0.022f_{y}d_{b}\right)+0.13{\left(\frac{2r}{b}+0.2\right)}^{0.1}\left(e^{-1.5\lambda_{f}}-e^{-40\lambda_{f}}\right)L$$

The first term $L_{P0}=\left(0.08L+0.22f_{y}d_{b}\right)$ is the plastic hinge length for unconfined RC columns, proposed by Paulay and Priestley [49]. While the rest of the Equation represents the effect of the FRP jacketing on the equivalent plastic hinge length; where r and b are corner radius and width of rectangular RC cross-sections, respectively.

To calculate the plastic hinge length, ${\;L}_{P}$ for the circular cross-section bridge columns, the Gu et al. [50] equation was used; as follows:

(2) $$L_{P}=\left(0.592-{2.30\lambda }_{f}+{2.28\lambda }_{f}^{2}\right)L+{0.022f}_{y}d_{b}\;\;\mathrm{When}\;\lambda_{f}>\;0.1$$

where, $\lambda_{f}$, $f_{y}$, and $d_{b}$ are the confinement ratio of FRP jackets, yield stress of the longitudinal reinforcement, and diameter of the longitudinal reinforcement, respectively. The confinement ratio of FRP jackets $\lambda_{f}$, is defined as $\lambda_{f\;}{=f}_{l,a}/{\overset{´}{f}}_{c}$, where $f_{l,a}$ is the actual confining pressure and ${\overset{´}{f}}_{c}$ is concrete compressive strength. Based on Lam and Teng [51], the actual confining pressure is determined as:

(3) $$f_{l,a}=\frac{{2E}_{frp}{t\varepsilon }_{h,rup}}{d}$$

where $E_{frp}$ is the elastic modulus of FRP jacket, t is the total thickness of FRP jacket,$\;\varepsilon_{h,rup}$ is hoop train at FRP rupture, and d is the diameter of core section. Based on Lam and Teng [51], $\varepsilon_{h,rup}$ is lower than ultimate tensile strain of FRP $\varepsilon_{frp}$ and equal to $\varepsilon_{h,rup}={0.586\varepsilon }_{frp}$.

### 4.3. Fiber Element Model

**Figure 4:**
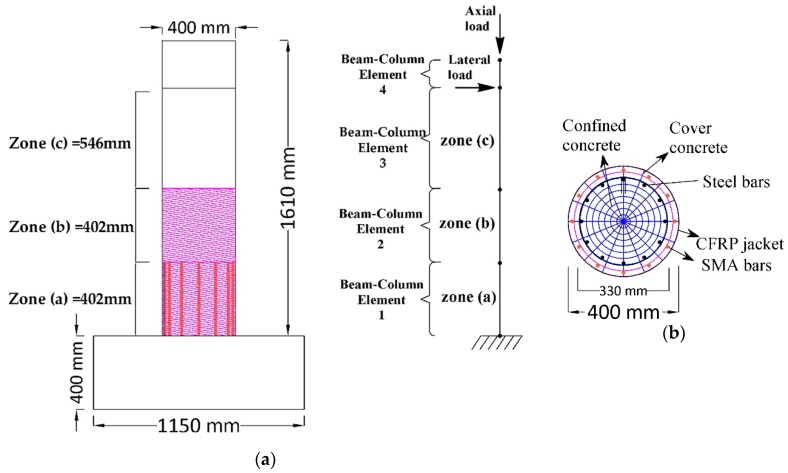
(**a**) Schematic of the column in Group1 and its numerical model; (**b**) fiber discretization of the retrofitted column in Group1.

**Figure 5:**
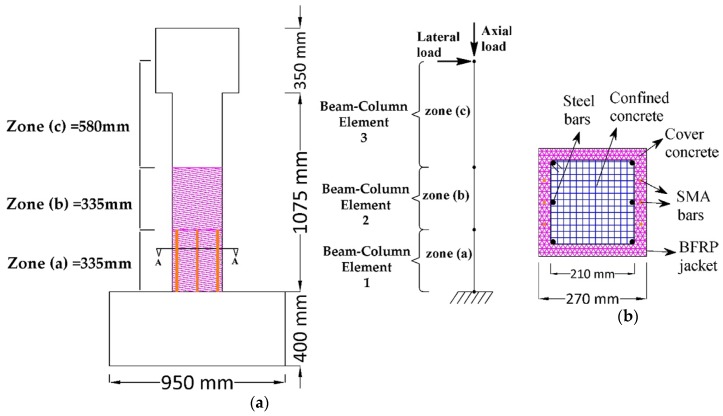
(**a**) Schematic of the column in Group2 and its numerical model; (**b**) fiber discretization of the retrofitted column in Group2.

The finite element program *OpenSees*, which has been specifically created for seismic analysis and earthquake simulations, has been employed to develop the analytical models of all the columns in the two groups. All the bridge columns were modeled with two dimensional (2D) nonlinear fiber beam-column elements. Since the two groups of columns have different geometric properties, two analytical models were developed by using *OpenSees*. The models used were cantilever columns subjected to lateral and axial loads. In the first analytical model, which described the circular cross-section columns, four force-based elements were used, see Figure 4a. While in the second model, the rectangular cross-section columns were divided into three nonlinear force-based elements, as illustrated in Figure 5a. For the two models, the fiber section was assigned to the beam-column elements to describe their nonlinear behavior. Figure 4b and Figure 5b show the fiber cross-section of the two analytical models. P-Delta effects were included in the analyses. To investigate the efficiency of the proposed retrofitting technique in improving the seismic behavior of RC bridge column, nonlinear static pushover, and reverse cyclic analyses were carried out in this study.

### 4.4. Description of Uniaxial Material Models

#### 4.4.1. Concrete

*OpenSees* has many material models describing the monotonic and cyclic response of concrete and steel reinforcement fibers. In this research; for unconfined cover concrete in all columns, the Kent-Scott-Park concrete model was used and assigned to the *Concrete01* model [52]. The confined concrete was modeled with the BGL model [53], which can be applied for different confinement kinds such as transverse reinforcement and/or external FRP warps. The BGL model was implemented in *OpenSees* and named *ConfinedConcrete01* [54].

#### 4.4.2. Reinforcing Steel 

For longitudinal reinforcing bars, the Giuffre–Menegotto–Pinto steel model [55] with isotropic strain hardening is adopted for the stress-strain relationship. This material model is implemented as *Steel02* in *OpenSees*. This material can represent nonlinear transition from the elastic stage to the strain hardening stage.

#### 4.4.3. NSM-SMA Bars 

*SelfCentering* material model, which was developed and implemented in OpenSees by Christopoulos et al. [56] was used to model superelastic NSM-SMA bars at the retrofitted zone (a). While, the shape memory effect NSM-SMA (FeMnSi) bar was modeled using *Hysteretic* material model. Moreover, based on investigations by Daghash and Ozbulut [57], mechanical anchorages of NSM-SMA bars were assumed. Therefore, the effect of the debonding of the NSM-SMA bars in all numerical models has been ignored. 

The material properties used in the analytical models of concrete and steel of all bridge columns are summarized in Table 6, while the mechanical properties of SMA bars were earlier mentioned in Table 1.

### 4.5. Validation of Numerical Model

**Figure 6:**
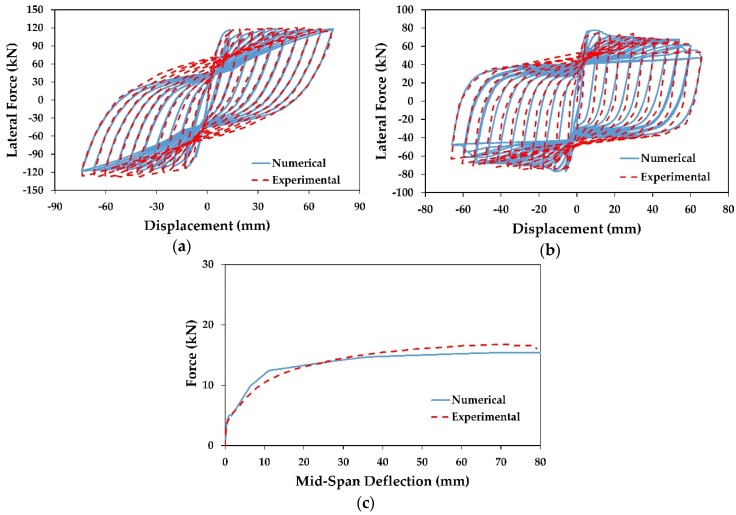
Comparison between the numerical and experimental results: (**a**) B2 column; (**b**) C2-0-3 column, (**c**) beam No. 2.

Validation and calibration of the numerical model are very essential to get realistic results. In this paper, experimental studies by Ding et al. [15] and Kawashima et al. [45] on scaled RC bridge columns were used to validate the numerical models. Figure 6a shows the comparison between the hysteretic curve of the experimental study of Kawashima et al. [45] conducted on a scaled RC bridge column wrapped with one layer of CFRP (column B2) and the numerical column model; while in Figure 6b, the hysteretic curve of the numerical model is compared with the C2-0-3 column tested by Ding et al. [15]. In general, the two numerical models showed a reasonable accuracy and were able to capture the behavior, specifically in the initial stiffness, lateral force capacity and loading and unloading paths. Moreover, based on the calibration results, the effect of bond-slip of the reinforcement steel bars was found to be negligible and the assumption of the full bond between the concrete and longitudinal bars was acceptable.

To the knowledge of the authors, there is no experimental or analytical study about retrofitting RC columns with NSM-SMA technique; therefore, the experimental study of strengthening RC beams using NSM- Fe-based SMA (beam No. 2) obtained by Shahverdi et al. [27] was used to validate NSM-SMA numerical models. From Figure 6c, it is evident that the numerical results are very close to the previous experimental results; and the numerical results varied only 10% in predicting the load-carrying capacity compared to those of the experimental results.

## 5. Static Pushover Analysis

**Figure 7:**
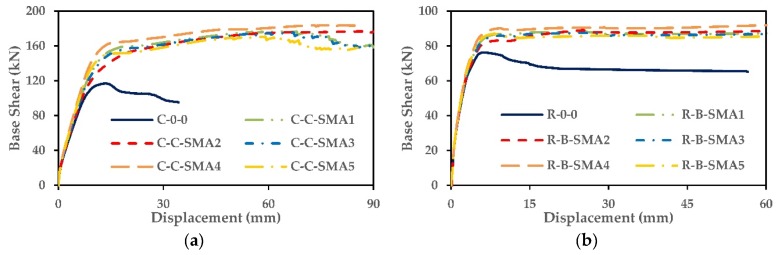
Pushover curves: (**a**) Columns of Group1; (**b**) columns of Group2.

Nonlinear static pushover analysis has been performed on each of the two columns groups. Two lumped masses 188 kN and 206 kN, which represented loads of the girders, were applied on the top of circular and rectangular RC bridges columns, respectively. The pushover curves for the two groups of columns are illustrated in Figure 7.

From Figure 7, it can be observed that, for the two groups, the proposed retrofitting columns increased deformation capacity compared with as-built columns considerably. The columns C-C-SMA2 and R-B-SMA2 showed the capability to undergo large displacements in Group1 and Group2, respectively. This is due to the fact that the FeNCATB alloy possesses the highest strain plateau and post-yield stiffness among other alloys. Since the retrofitted columns in each group showed similar base shear capacity, the retrofitted columns were compared in terms of displacement ductility capacity.

### 5.1. Displacement Ductility

**Figure 8:**
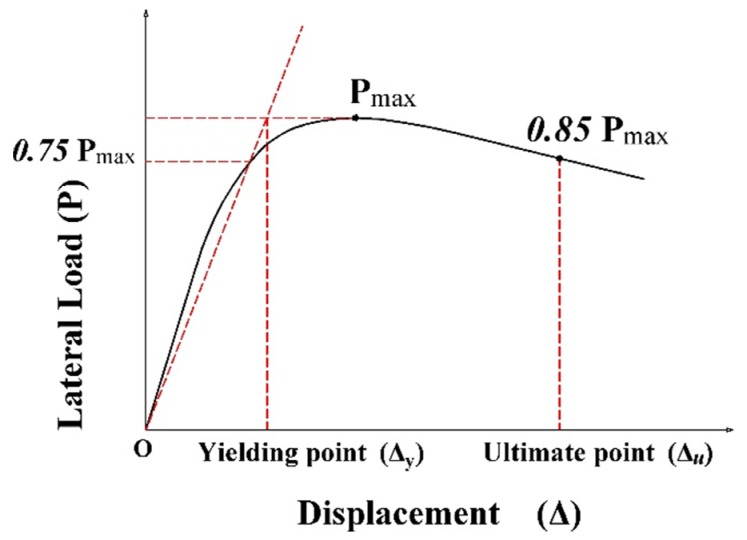
Define yield and ultimate displacement.

From the pushover analysis, the displacement ductility $\mu_{\Delta }$ was calculated for all columns in the two groups. The displacement ductility is defined as $\mu_{\Delta }=\Delta_{u}/\Delta_{y}$, where $\Delta_{y}$ is the yield displacement of the column and $\Delta_{u}$ is the ultimate displacement of the column. The yield and ultimate displacement can be estimated by different methods [49,58,59]. In this paper, the method proposed by Park [59] was used. From Figure 8, the $\Delta_{y}$ is defined from the equivalent elastoplastic curve as the intersection point between the secant stiffness line at 75% of maximum lateral load $P_{max}$ and the horizontal $P_{max}$ line. $\Delta_{u}$ is the displacement corresponded to 85% of maximum lateral load $P_{max}$, or when the strain of the longitudinal reinforcement reaches to the fracture strain, whichever occurs first [59]. 

**Figure 9:**
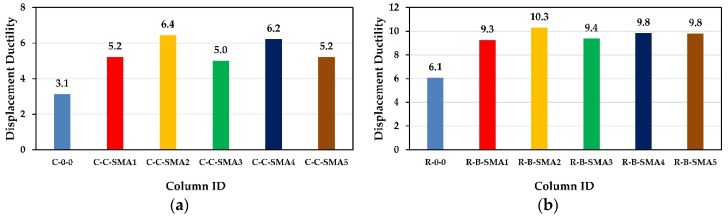
Displacement ductility capacity: (**a**) Columns of Group1; (**b**) columns of Group2.

A comparison of displacement ductility of all retrofitted RC columns in the two groups is shown in Figure 9. According to this figure, the displacement ductility of all retrofitted columns in the two groups was remarkably higher than that of as-built columns. 

From Figure 9a, it is clear that the displacement ductility of the column C-C-SMA2 was 6.4, which was 23%, 28%, 3%, and 23% higher than that of C-C-SMA1, C-C-SMA3, C-C-SMA4, and C-C-SMA5, respectively. Moreover, the results showed that the columns C-C-SMA3 provide the minimum displacement ductility of 5.0 among other retrofitted columns in Group1. While for Group2, the maximum displacement ductility of 10.3 belonged to the column R-B-SMA2. The difference between retrofitted columns in Group2 in terms of displacement ductility was very small. The minimum displacement ductility was for the column R-B-SMA1 which 52% higher than that of R-0-0.

#### 5.1.1. Discussion of the Displacement Ductility

It was noticed from Figure 9 that the columns retrofitted with NSM-FeNCATB were able to undergo the larger displacements than other retrofitted columns; therefore, these columns had the maximum displacement ductility among other columns. This is due to the face that the FeNCATB alloy possesses the highest strain plateau and post-yield stiffness among other alloys. On the other hand, the columns C-C-SMA4 and R-B-SMA4 showed high displacement ductility in Group1 and Group2, separately. This is because FeMnAlNi alloy has very low yield strength to elastic modulus ratio (0.0033); and consequently, the columns C-C-SMA4 and R-B-SMA4 yielded at small displacements. 

#### 5.1.2. Discussion of the Effect of NSM-SMA Bars on The Yield Displacement

Based on the aforementioned results, the retrofitted columns in Group1 and Group2 exhibited higher ductility compared with as-built columns. The question here is how the NSM-SMA bars can affect the displacement ductility of the retrofitted columns with the proposed retrofitting technique in this paper.

**Figure 10:**
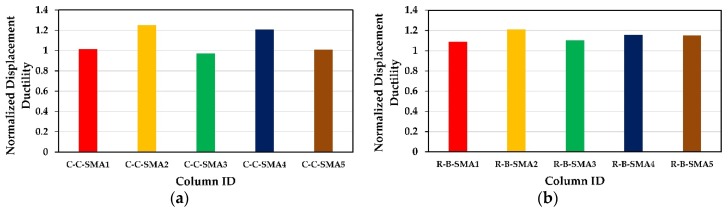
Normalized displacement ductility: (**a**) Columns of Group1; (**b**) columns of Group2.

Figure 10 can answer this question; in which it presents the retrofitted columns versus the normalized displacement ductility for Group1 and Group2, separately. The normalized displacement ductility is defined as the displacement ductility of each of the retrofitted columns of the two groups divided by the ductility of the columns retrofitted with FRP jackets only (C-C-0 and R-B-0). The displacement ductility of the columns C-C-0 and R-B-0 were calculated to be 5.2 and 8.5, respectively. From Figure 10a, it is evident that for Group1, the displacement ductility of the columns C-C-SMA1, C-C-SMA3, and C-C-SMA5 was almost equal to that of the column C-C-0 (CFRP jacket only). However, the columns C-C-SMA2 and C-C-SMA4 showed good enhancements in displacement ductility which were 25% and 21% higher than that of the column C-C-0. On the other hand, according to Figure 10b, all retrofitted columns in Group2 showed an improvement in the displacement ductility compared with R-B-0 (CFRP jacket only). This improvement was about 9% for columns R-B-SMA1 and R-B-SMA3, leading to about 21, 16, and 15% for columns R-B-SMA2, R-B-SMA4, and R-B-SMA5, respectively. 

**Figure 11:**
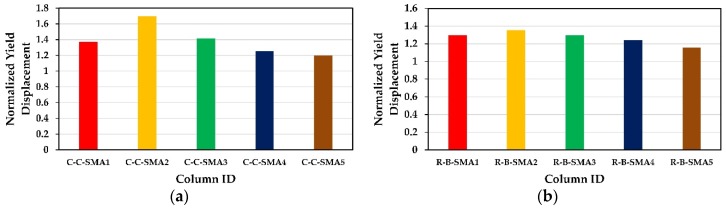
Normalized yield displacement: (**a**) Columns of Group1; (**b**) columns of Group2.

It is worth noticing that, in the two groups, the NSM-NiTi and CuAlNi bars did not significantly improve the displacement ductility of the retrofitted columns. Moreover, the FRP jacketing (CFRP or BFRP) was the most effective in increasing the displacement ductility; whereas the effect of Fe-based SMAs (FeNCATB, FeMnAlNi, and FeMnSi) in improving the displacement ductility was more obvious. This is attributed to the low post yielding strength and the high yield strength to elastic modulus ratio of NiTi (0.0064) and CuAlNi (0.0075) alloys; i.e., the columns retrofitted with these two alloys cannot undergo large displacements but at the same time have good yield displacement as shown in Figure 11.

Figure 11 presents the retrofitted columns versus normalized yield displacement is yield displacement for Group1 and Group2, separately. The normalized yield displacement is defined as the yield displacement of each of the retrofitted columns of the two groups divided by the yield displacement of the columns retrofitted with FRP jackets only. 

It is found from Figure 11 that the NSM-SMA bars had increased the yield displacement remarkably compared to the FRP (CFRP or BFRP) wrapped columns in the two groups. This increase for Group1 was varying between 20% of the column C-C-SMA5 and 70% of the column C-C-SMA2. It was found also the yield displacement of the column C-C-SMA2 was 38% greater than that of the column C-C-SMA4. While for Group2, the yield displacement of the columns R-B-SMA2 was 36% higher than that of the column R-B-0. In general, the increase in yield displacement plays an important role in reducing the ductility demand of the retrofitted columns. Therefore, the proposed technique can be effectively used to retrofit RC bridge columns in the seismic zones. 

## 6. Cyclic Loading Analysis

**Figure 12:**
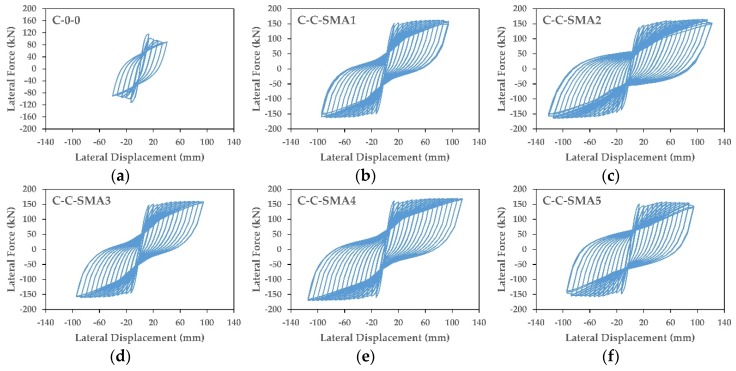
(**a**–**f**) Force displacement relationship of the columns in Group1.

**Figure 13:**
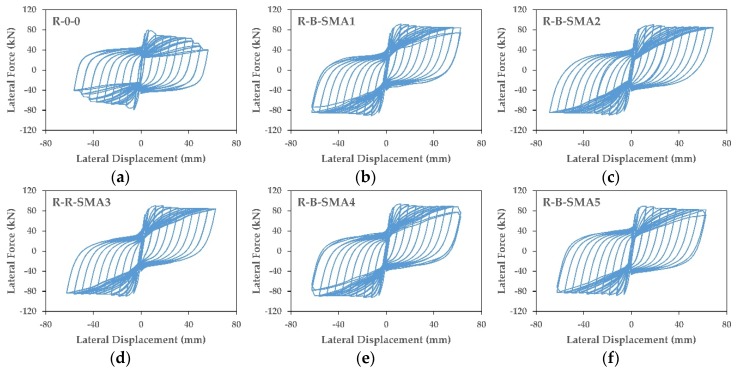
(**a**–**f**) Force displacement relationship of the columns in Group2.

Displacement controlled-quasi-static cyclic analysis was applied to all the columns. Three full cycles were applied to the studied columns at each drift ratio with an increment of 0.5% drift. Figure 12 and Figure 13 present the hysteretic behavior of retrofitted columns in Group1 and Group2, respectively. It can be observed that the retrofitted columns in the two groups showed higher and more stable load-carrying capacity compared to the as-built columns.

For the retrofitted columns in Group1, the results showed that the ultimate lateral force for the column C-C-SMA1 was 161.3 kN at a drift ratio of 7%; whereas for the columns C-C-SMA2, C-C-SMA3, C-C-SMA4, and C-C-SMA5 was 164.8, 159.3, 167.6, and 158.3 kN at drift ratios of 9%, 7%, 8.5%, and 7%, respectively. It is clear that the column C-C-SMA2 had the ability to undergo to the maximum imposed drift of 9% with good lateral force. This is attributed to the high ultimate strength of the FeNCATB alloy. While for Group2, the ultimate lateral force of the column R-B-SMA2 was 85.1 kN at the drift ratio of 5.5%; whereas, other retrofitted columns did not exceed the drift ratio of 5%.

### 6.1. Relative Self Centering

In this section, the self-centering capacity of the retrofitted columns is discussed. Based on Sideris et al. [60] relation, the Relative Self Centering Efficiency RSE represents the recoverability percentage of the peak displacement at each cycle and is defined as follows:

(4) $$\mathrm{RSE}=1-\frac{u_{res}^{+}-u_{res}^{-}}{u_{Peak}^{+}-u_{Peak}^{-}}$$

**Figure 14:**
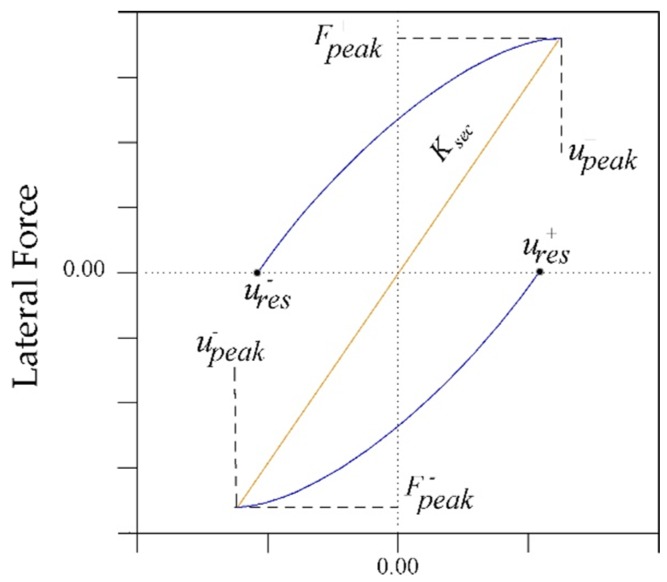
Peak and residual displacement in a single cycle.

As shown in Figure 14, $u_{res}^{+}$, $u_{res}^{-}$, $u_{Peak}^{+}$, and $u_{Peak}^{-}$ refer to the positive and negative residual displacements (average of three cycles), the positive and negative peak displacements, respectively.

**Figure 15:**
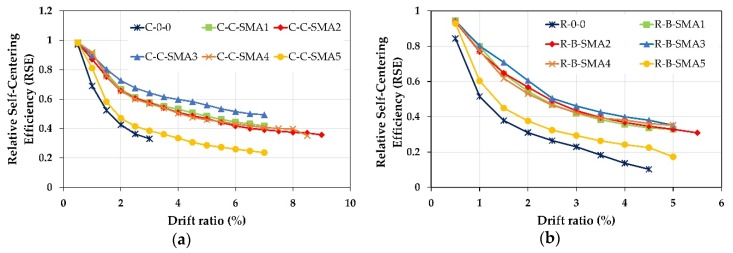
Relative Self Centering Efficiency versus the drift ratio: (**a**) Columns of Group1; (**b**) columns of Group2.

It is clear from Figure 15 that for the two groups, the RC columns retrofitted with superelastic SMA bars reported better self-centering performance than as-built columns. In addition, it is noticed that the columns retrofitted with NSM-CuAlNi and NSM-FeMnSi bars provided the best and worst self-centering capability among the other retrofitted columns. 

**Figure 16:**
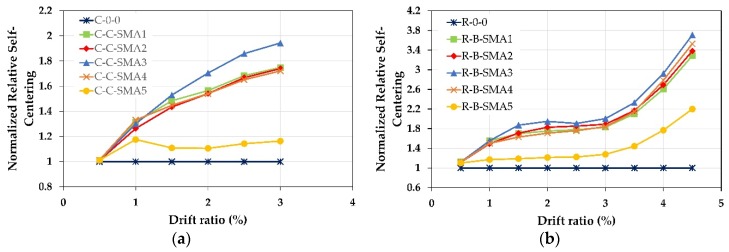
Normalized Relative Self Centering Efficiency versus the drift ratio: (**a**) columns of Group1; (**b**) columns of Group2.

Figure 16 shows the efficiency of the proposed retrofitting technique in increasing the self-centering capability of the RC column; where the drift ratios versus normalized relative self-centering RSE ratio for the two groups is illustrated. The normalized relative self-centering is equal to the relative self-centering ratio of each retrofitted column divided by the relative self-centering ratio of the as-built columns.

#### Discussion of the Relative Self-Centering

For Group1, up to the drift ratio 1.5% all retrofitted RC columns except the column C-C-SMA5 had almost the same RSE values. When the column C-0-0 failed at the drift ratio of 3%, the RSE of C-C-SMA3 was 0.64; i.e., 64% of the peak displacement could be recovered, while this percentage drops to 39% and 33% for the columns C-C-SMA5 and C-0-0, respectively. This is because the FeMnSi alloys do not have superelastic behavior. Moreover, it is observed that the NSM-FeNCATB and NSM-FeMnAlNi bars had almost the same self-centering performance; however, the diameter of the NSM-FeNCATB bars used was much smaller than that of NSM-FeMnAlNi bars. On the other hand, for Group2, at the drift ratio of 1%, approximately 80% of the peak deformation was recovered for the columns retrofitted with superelastic SMA bars. It is noticed that up to the drift ratio of 3% the performance of the columns R-B-SMA2 was better than that of the column R-B-SMA4.

Figure 16a demonstrated that at a small drift ratio of 0.5% the relative self-centering of all retrofitted was equal to column C-0-0. This is because the NSM-SMA bars are distributed on the circumference of the circular cross-section column and only a few NSM-SMA bars contributed to reducing the permanent deformation. The RSE of the columns C-C-SMA3, C-C-SMA1, C-C-SMA2, and C-C-SMA4 was 90%, 77%, 75%, and 72% better than C-0-0. While the column C-C-SMA5 could not reduce the residual deformation of more than 20%. While for Group2, (rectangular cross-section), all NSM-SMA bars deformed at the same time. Therefore, the proposed retrofit technique was more effective in reducing residual deformation. From Figure 16b, for all retrofitted columns except the column R-B-SMA5, it can be seen that up to a drift ratio of 2%, just approximately 80% of peak displacement could be recovered. However, beyond that drift ratio, the contribution of NSM-SMA bars increased, therefore the RSE was increased significantly, and reached for the column R-B-SMA3 about 3.7 times that of the R-0-0 column.

### 6.2. Equivalent Viscous Damping Ratio

**Figure 17:**
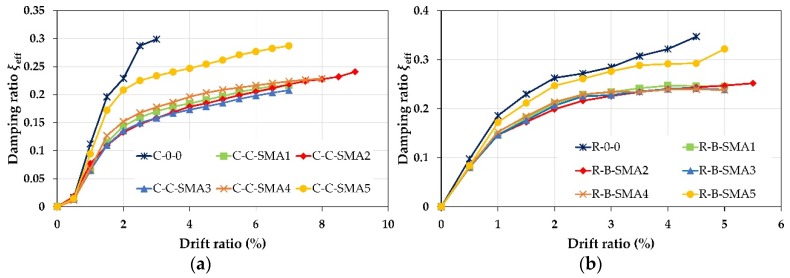
Equivalent viscous damping ratio: (**a**) Columns of Group1; (**b**) columns of Group2.

The equivalent viscous damping ratio was used to evaluate the energy dissipation capacity of RC columns retrofitted with the proposed technique in this paper. Based on Sideris et al. [60], the equivalent viscous damping ratio $\xi_{eff}$, for one given cycle, is defined as follows:

(5) $$\xi_{eff}=\frac{E_{d}}{{2\pi K}_{sec}u_{o}^{2}}$$

where $E_{d}$ is the total energy dissipated per cycle (average of three cycles), $K_{sec}$ is the secant stiffness, and $u_{o}$ is the amplitude of the cycle. The Equivalent damping ratio is calculated and the results is illustrated in Figure 17.

From Figure 17, the columns retrofitted with NSM-FeMnSi bars showed the closest equivalent viscous damping ratio to as-built columns in the two groups. On the contrary, among all the retrofitted columns, the columns C-C-SMA3 and R-B-SMA3 provided the lowest performance in Group1 and Group2, separately. On the other hand, the columns retrofitted with NSM-FeMnAlNi bars showed better performance among the superelastic NSM-SMA bars.

#### Discussion of the Damping Ratio

It was found from Figure 17 that the larger self-centering happened the lower damping ratio generated. This is because the flag-shaped behavior on the NSM-SMA bars controlled the hysteretic curves of the retrofitted columns. Thus, the area of loops became smaller leading to lower energy dissipation.

It is obvious from Figure 17a that, among the columns retrofitted with superelastic NSM-SMAs, the column C-C-SMA4 had better equivalent viscous damping up to drift ratio of 7.0%, beyond that drift the dissipated energy of this column was similar to the column C-C-SMA2. However, due to high deformability of NSM-FeNCATB bars, the column C-C-SMA2 dissipated more energy than other columns after the drift ratio of 7%; while, for Group2, see Figure 17b, the equivalent viscous damping ratio $\xi_{eff}$ of the column R-B-SMA4 was 23% at a drift ratio of 3.0%, after this drift ratio the column R-B-SMA2 showed a better energy dissipation capacity.

## 7. Parametric Study 

It was found from the aforementioned results that among the retrofitted columns in the two groups, the columns retrofitted with NSM-FeNCATB bars and FRP jackets showed superior performance in terms of displacement ductility and large deformation capacity. Moreover, despite its small diameter, the NSM-FeNCATB bars exhibited good self-centering capability at high drift ratios. This makes using FeNCATB as NSM reinforcement bars a powerful potential candidate in retrofitting RC bridge columns. This result is similar to that obtained by Billah and Alam [30,61]. Therefore, the columns C-C-SMA2 and R-B-SMA2 were selected in this numerical parametric to investigate some basic parameters that affect the behavior of retrofitted columns. These parameters include the NSM-SMA ratio $\rho_{NSM}$, and number of FRP (CFRP or BFRP) jackets.

### 7.1. Effect of NSM-SMA Bar Size

**Figure 18:**
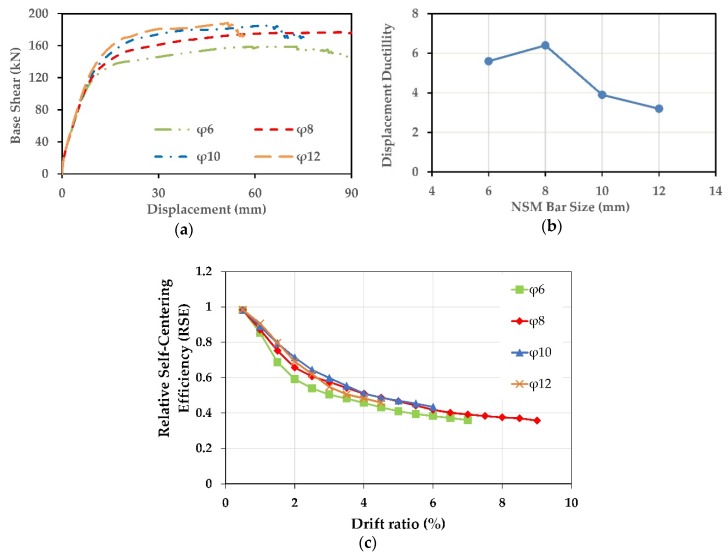
Effect of NSM-SMA bar size on the column C-C-SMA2: (**a**) Pushover curves; (**b**) displacement ductility; (**c**) Relative Self Centering Efficiency.

The effect of the amount of the NSM-FeNCATB bars on the performance of the columns C-C-SMA2 and R-B-SMA2 was investigated in this section. Four sizes of SMA bars (φ6, φ8, Φ10, and φ12) were selected. The geometric ratios of NSM reinforcements,$\;\rho_{NSM}$ equal to 0.27%, 0.48%, 0.75%, and 1.08% for the column C-C-SMA2 and 0.23%, 0.41%, 0.65%, and 0.93% for the column R-B-SMA2, respectively. The location and spacing of the NSM-FeNCATB bars were kept unchanged. The other properties and the number of FRP jacket layers of both columns were also kept unchanged. Figure 18 illustrates the effect of the NSM-SMA bar size on the column C-C-SMA2.

Figure 18a shows the pushover curves of the C-C-SMA2 for each NSM bar size. As expected, the base shear of the C-C-SMA2 column increased by increasing the NSM bar size. However, the main objective of this proposed retrofitting technique is improving the ductility capacity and mitigating the residual displacement of the bridge columns. From Figure 18b, it is clear that the maximum displacement ductility obtained when the RC column retrofitted with φ8 NSM-SMA bars. Moreover, increasing the size of the NSM bar up to 10 and 12 mm lead to a little reduction in the displacement ductility. 

**Figure 19:**
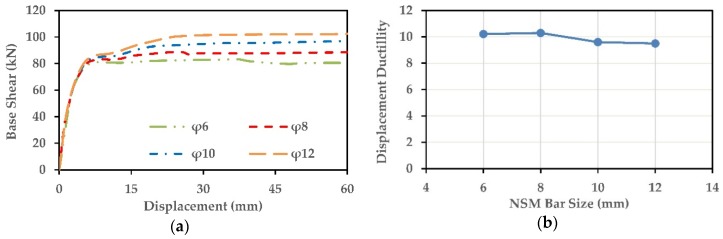

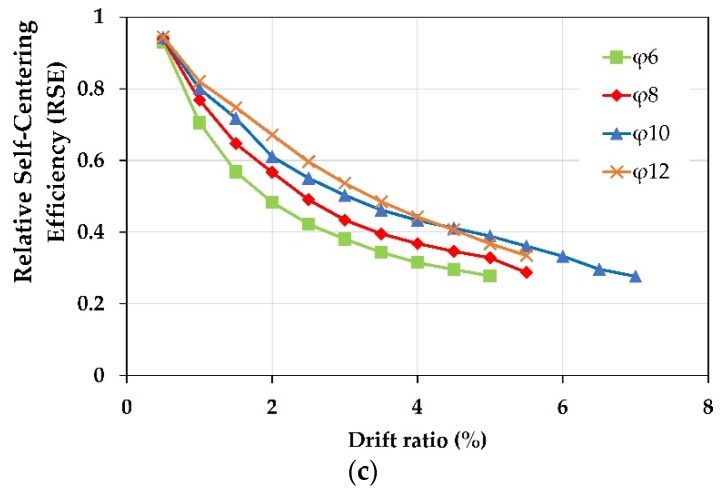
Effect of NSM-SMA bar size on the column R-B-SMA2: (**a**) Pushover curves; (**b**) displacement ductility; (**c**) Relative Self Centering Efficiency.

Changing the size of NSM bar also affected the self-centering capability of the column C-C-SMA2. As shown in Figure 18c, the relative self-centering capacity improved by increasing the NSM-SMA bar size. It was also noticed that at high NSM-bar sizes of φ10 and φ12, the C-C-SMA2 failed at the drift ratios of 6% and 4.5%, respectively. Moreover, the high NSM-bar sizes did not provide more self-centering than that obtained when the column C-C-SMA2 retrofitted with φ8 NSM bars. This weak performance is attributed to the circular geometry of the column C-C-SMA2 where the NSM bars were embedded in the circumference of the column. Figure 19 illustrates the effect of the NSM-SMA bar size on the column R-B-SMA2.

For the column R-B-SMA2, as shown in Figure 19a, the pushover curves were more stable by increasing the NSM bar size. Similar to the column C-C-SMA2, the column R-B-SMA2 retrofitted with φ8 NSM-SMA bars showed better displacement ductility. However, by changing the NSM bar size, the difference in displacement ductility was lower 5%. In another word for this column, the displacement ductility had been not affected by the amount of the NSM-SMA bars. According to Figure 19c, the relative self-centering of the R-B-SMA2 column was highly improved by increasing the NSM bar size. However, increasing the NSM bar size up to 12mm did not improve the self-centering after the drift ratio of 4.5%. 

### 7.2. Effect of Number of FRP Layer 

**Figure 20:**
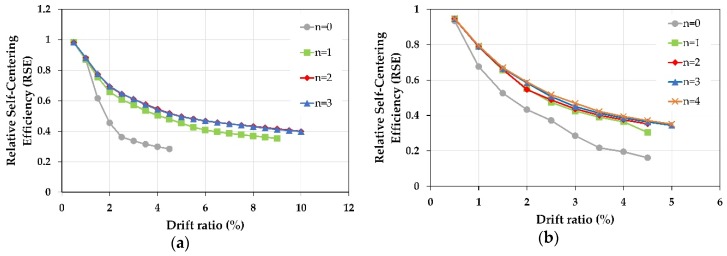
Effect of number of FRP layers: (**a**) The column C-C-SMA2; (**b**) the column R-B-SMA2.

As known, the FRP jacketing has a positive effect on the ductility of the RC columns. Therefore, in this section, only the effect of the number of FRP layers on the self-centering capacity of the retrofitted columns C-C-SMA2 and R-B-SMA2 was discussed. Thus, the number of FRP layers “n” in the two columns was changed from n = 1 to n = 4. The mechanical properties of concrete, steel, and NSM-SMA bars remained unchanged. In order to study the effect of changing FRP layer number only on the self-centering capacity, the length of the critical zone (a) and zone (b) was assumed equal to 400 mm (diameter of the cross-section) and 270 mm (depth of the cross-section) for the column C-C-SMA2 and R-B-SMA2, respectively. Figure 20 illustrates the relative self-centering efficiency of the columns C-C-SMA2 and R-B-SMA2 retrofitted with NSM-FeNCATB bars and different numbers of FRP jackets.

According to the above figure, using at least one layer of FRP jacketing improved the self-centering capability by 60% and 65% of the columns C-C-SMA2 and R-B-SMA2, respectively. This is because FRP wrapping of columns prevents buckling and debonding of NSM-SMA bars. It is clear from Figure 20a, by increasing the number of CFRP jackets from n = 2 to n = 4 the RSE curves were very similar to each other. On the other hand, from Figure 20b, for the column R-B-SMA2, the RSE curves showed roughly the same behavior when the number of BFRP layers changed from n = 1 to n = 4, but when three and four layers of BFRP jackets were used the column sustained larger lateral drifts. The optimum number of FRP layers was 2 for and 3 for the column C-C-SMA2 and R-B-SMA2, separately. In general, using FRP jacketing plays an important role in the effectiveness of this proposed retrofit technique. 

## 8. Comparison to an NSM FRP Strengthened RC Column

**Figure 21:**
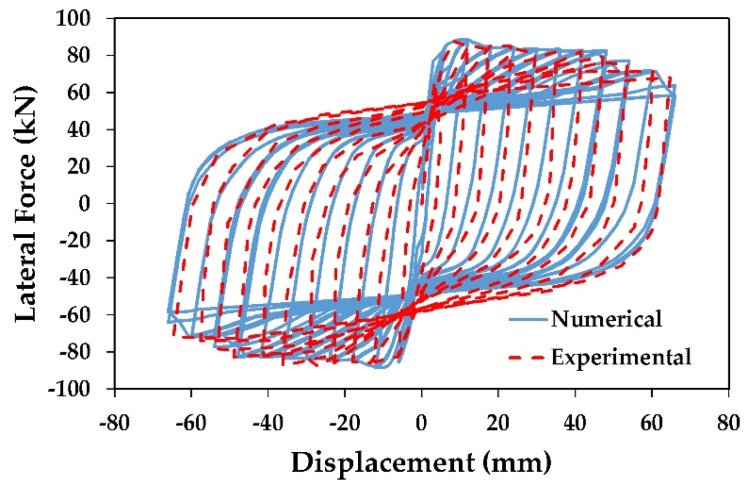
Comparison of the numerical and experimental hysteretic curve of the column C3-6-3 tested by Ding et al. [15].

In this section, the efficiency of the proposed retrofitting technique in this paper was compared with NSM BFRP retrofitting approach. For this purpose, the column R-B-SMA2 was selected. The mechanical properties of the concrete and steel were similar to those given in Section 4.3. the NSM-FeNCATB bars were replaced with NSM-BFRP bars in such a way that the column retrofitted with NSM-BFRP bars (named R-B-BFRP) had almost the same axial force of the column R-B-SMA2. Therefore, the number of NSM-BFRP bars remained 3 and the diameter of NSM-BFRP bars was taken 6 mm. the number of FRP jacket layers in the column R-B-BFRP was also kept unchanged. To validate NSM-BFRP numerical model, the experimental study by Ding et al. [15] on the column C3-6-3 was used. Figure 21 demonstrates a good agreement between the numerical and experimental results.

**Figure 22:**
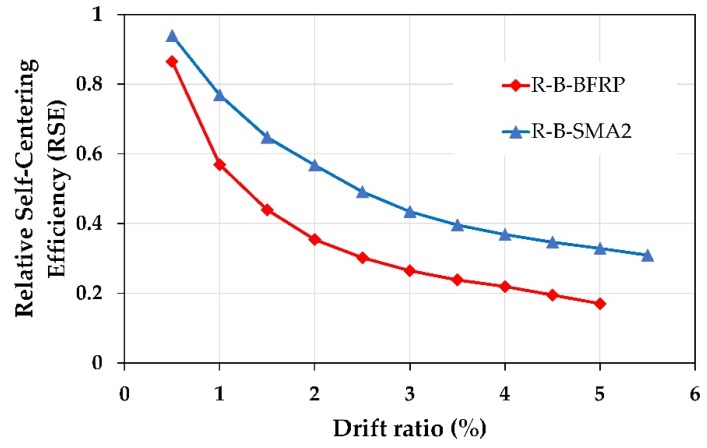
Relative self-centering efficiency of the columns R-B-SMA2 and R-B-BFRP.

The comparison of self-centering capacity of the columns R-B-SMA2 and R-B-BFRP is shown in Figure 22. It can be observed that the relative self-centering of the column retrofitted with NSM-FeNCATB bars was approximately 80% higher than that of the column NSM-BFRP. 

## 9. Conclusions

This analytical study investigated the application of an innovative retrofit technique for RC bridge columns by using five different types of NSM-SMA bars with FRP jackets. In this retrofitting technique, the RC bridge column was divided into three zones. The first zone where the plastic hinge was expected to occur, was retrofitted with NSM-SMA bars and FRP jackets. The second zone was retrofitted with FRP jacket only; and the rest of the column (third zone) was kept without any retrofitting. One rectangular and one circular RC bridge columns were retrofitted with 5 types of SMAs and wrapped with FRP jacket. The performance of the above RC columns was numerically investigated subjected to nonlinear static and lateral cyclic loading via *OpenSees* software.

Based on the numerical investigation, the most important conclusions are summarized as follows:

(1) All retrofitted columns with this proposed technique exhibited improvement in displacement ductility capacity compared with as-built columns. Among all retrofitted columns the columns C-C-SMA2 and R-B-SMA2 showed greater deformation capacity and displacement ductility.
(2) The columns retrofitted with CuAlMn and NiTi and FRP jacket alloys did not show an enhancement in displacement ductility compared with those retrofitted with FRP jacket only.
(3) This study indicated that the NSM-SMA bars had increased the yield displacement of the RC columns remarkably. This plays an important role in reducing the ductility demand of the RC bridge columns in the seismic zones.
(4) At the same lateral strength level, as expected, the columns retrofitted with NSM-FeMnSi bars did not reduce the residual displacement. This is because this alloy does not have superelastic behavior. On the other hand, the columns retrofitted with NSM-CuAlNi bars showed better self-centering capacity.
(5) In general, the efficiency of this proposed retrofitting technique in improving self-centering capacity was better in rectangular cross-section columns than circular cross-section ones. This is because the NSM-SMA bars were distributed along the circumference of the circular columns.
(6) The columns retrofitted with NSM-FeMnSi bars and FRP jackets dissipated higher amounts of energy than other retrofitted columns. It is found also that the larger self-centering happened the lower damping ratio generated. This is attributed to the superelastic behavior of NSM-SMA bars.
(7) Increasing the NSM-FeNCATB bar size decreased the displacement ductility of the column C-C-SMA2 and at the same time did not improve the self-centering capacity than that obtained when the column C-C-SMA2 retrofitted with φ8 NSM bar. While for the column R-B-SMA2, the relative self-centering of the R-B-SMA2 column was highly improved by increasing the NSM bar size.
(8) It was found also, the FRP jackets played an important role in the enhancement of the effect of this proposed technique. However, increasing the number of FRP up a certain number did not affect the self-centering capacity of the RC columns.
(9) Compared with NSM-BFRP, the proposed retrofitting technique showed better performance in terms self-centering capability.

## Figures and Tables

**Table 1:** Properties of different types of Shape Memory Alloys (SMAs).

Alloy	$\varepsilon_{s}$ (%)	*E* (Gpa)	$f_{y}$ (Mpa)	$f_{p1}$ (Mpa)	$f_{T1}$ (Mpa)	$f_{T2}$ (Mpa)	fyE	Reference
NiTi45	8	68	435	535	335	170	0.0064	Ghassemieh et al. [43]
FeNCATB	13.5	46.9	750	1200	300	200	0.0160	Tanaka et al. [33]
CuAlMn	9	28	210	275	200	150	0.0075	Shrestha et al. [44]
FeMnAlNi	6.13	98.4	320	442.5	210	122	0.0033	Omori et al. [34]

$\varepsilon_{s}$: superelastic plateau strain length, *E*: Young’s modulus, $f_{y}$: austenite to Martensite starting stress, $f_{p1}$: Austenite to Martensite finishing stress, $f_{T1}$: Martensite to austenite starting stress, $f_{T2}$: Martensite to austenite finishing stress.

**Table 2:** Material properties and dimensions of the two columns adopted in this study.

Bridge column Properties	Circular	Rectangular
Cross-section dimension (mm)	Diameter 400	270 × 270
Effective height of column (mm)	1350	1250
Longitudinal reinforcement ratio (%)	1.89	0.93
Volumetric ratio of lateral reinforcement (%)	0.128	1.60
Compressive strength of concrete (MPa)	30	29.65
Yield strength of longitudinal rebar (MPa)	374	465
Yield strength of transverse rebar (MPa)	363	342
Axial load (kN)	188	206

**Table 3:** Mechanical properties of the two types of Fiber-Reinforced Polymer (FRP) jackets.

FRP Properties	BFRP	CFRP
*n*	3	1
*t* (mm)	0.11	0.111
$f_{f}$(MPa)	1716	4476
$E_{f}$(GPa)	88	266

n: number of FRP wraps; *t*: thickness; $f_{f}$: tensile strength; $E_{f}$: elastic modulus

**Table 4:** As-built and retrofitted columns of the two groups.

	Column ID	Retrofitting Type
Group1	C-0-0	As-built, not retrofitted
	C-C-SMA1	retrofitted with CFRP jacket and NSM NiTi45 bars
	C-C-SMA2	retrofitted with CFRP jacket and NSM FeNCATB bars
	C-C-SMA3	retrofitted with CFRP jacket and NSM CuAlMn bars
	C-C-SMA4	retrofitted with CFRP jacket and NSM FeMnAlNi bars
	C-C-SMA5	retrofitted with CFRP jacket and NSM FeMnSi bars
Group2	R-0-0	As-built, not retrofitted
	R-B-SMA1	retrofitted with BFRP jacket and NSM NiTi45 bars
	R-B-SMA2	retrofitted with BFRP jacket and NSM FeNCATB bars
	R-B-SMA3	retrofitted with BFRP jacket and NSM CuAlMn bars
	R-B-SMA4	retrofitted with BFRP jacket and NSM FeMnAlNi bars
	R-B-SMA5	retrofitted with BFRP jacket and NSM FeMnSi bars

**Table 5:** Near-surface mounted (NSM) reinforcement ratio of at some researches.

Research	NSM Reinforcements Type	Steel Reinforcements Ratio $\rho_{s}$(%)	NSM Reinforcements Ratio $\rho_{NSM}$ (%)
Sarafraz [47]	GFRP bar	0.785	0.502–0.785–1.13
Barros et al. [14]	CFRP strips	0.785–1.13	0.25
Ding et al. [15]	BFRP bars	0.93	0.28-0.43-0.62
Seifi et al. [17]	GFRP bars	0.985	0.5
	steel bars	0.985	0.723

**Table 6:** Material properties used in the model.

Material Model	Property	Columns Group 1	Columns Group 2
Unconfined concrete	Compressive Strength (MPa)	30	29.65
	Strain at peak stress (%)	0.002	0.002
	Modulus of elasticity (MPa)	27380	27227
Confined concrete	Compressive strength (MPa)	32	36.47
	Strain at peak stress (%)	0.0025	0.004
	Modulus of elasticity (MPa)	27380	27227
Longitudinal steel	Young’s modulus (GPa)	149.6	214.15
	Yield Strength (MPa)	374	465
	Ultimate strength	420	594
	Yield strain (%)	0.0025	0.00217
